# Biofilm-associated antibiotic tolerance in the era of multidrug resistance: quorum-sensing mechanisms and emerging therapeutic strategies

**DOI:** 10.3389/fcimb.2026.1826282

**Published:** 2026-04-28

**Authors:** Tarunkarthick Subramani, Elizabeth Annie George, Akash Eldhose Saju, Muralikrishnan E., Ranit De, Siddharth A. K., Sudha Ramaiah, Anand Anbarasu

**Affiliations:** 1 Department of Biotechnology, School of Biosciences and Technology, Vellore Institute of Technology (VIT), Vellore, Tamil Nadu, India; 2 Department of Bio-Sciences, School of Biosciences and Technology, Vellore Institute of Technology (VIT), Vellore, Tamil Nadu, India; 3 Medical and Biological Computing Laboratory, School of Biosciences and Technology, Vellore Institute of Technology (VIT), Vellore, Tamil Nadu, India

**Keywords:** antimicrobial resistance, biofilm, extracellular matrix, peptides, quorum sensing

## Abstract

Biofilms are highly organised complex structures formed by microorganisms that adhere to surfaces and are embedded with an extracellular polymeric matrix. This matrix provides structural stability, retains nutrients and offers defence against unfavourable environments and antibiotics. Multi-layered molecular mechanisms controlled by quorum-sensing networks are involved in the transition from the planktonic stage to a mature biofilm. Surface attachment, maturation and dispersion are coordinated by these mechanisms, which also provide communication between different species. Biofilm development poses a significant challenge to implants in nosocomial settings and is considered a major threat in the global health care sector and industries, leading to persistent infection. In addition to assessing current biofilm management techniques such as quorum quenching agents, enzymatic matrix disruptions, antimicrobial peptides, nanoparticles and metal complex-based interventions, this review highlights the major regulatory components and molecular mechanisms causing biofilm formation. It also emphasises the necessity to combat biofilm-associated tolerance by highlighting the increasing significance of computational approaches in drug discovery and development of next-generation anti-biofilm therapeutics.

## 1 Introduction

One of the threats to world health in the 21^st^ century is still antimicrobial resistance (AMR). Turning deadly diseases into treatable conditions has led to the discovery and clinical application of antibiotics, enhancing the healthcare industry (Laxminarayan et al., 2016). However, the extensive and careless use of antibiotics in agriculture, animal husbandry, and human health has accelerated the evolution of microbial population resistance. 1.27 million fatalities in 2019 were directly caused by AMR, whilst an additional 4.97 million deaths were linked to medication resistance globally (Murray et al., 2022). One important factor exacerbating the AMR crisis is the bacteria’s ability to form biofilms. Biofilms are organised bacterial populations that are shielded from abiotic and biotic surfaces by an extracellular polymeric substance (EPS) that the microbes produce themselves (Flemming and Wingender, 2010). Biofilms exhibit a complex structure, incorporating channels for nutrient supply and quorum sensing, thereby making the bacteria highly tolerant to antibiotics and helping the persister cells (Lewis, 2010). It is important to distinguish biofilm-associated phenotypic tolerance from genetically inherited AMR, as the former is reversible and driven by physiological and environmental factors rather than stable genetic mutations, although biofilms can facilitate emergence and dissemination of genetic resistance through horizontal gene transfer. When biofilm cells differentiate and synchronise their life cycles, they create a heterogeneous environment. Emergent qualities of biofilm communities include the development of new structures, behavioural patterns, and functional characteristics that develop throughout the process of self-organisation (Panickar et al., 2024). Biofilm matrix contains up to 97% of water, and it comprises both soluble, gel-forming polysaccharides and extracellular DNA (eDNA), as well as insoluble elements such as amyloids, fimbriae, pili, and flagella, which contribute to the structural and functional components of the biofilm (Dutt et al., 2022).

Biofilms enter into dynamic phases that promote the spread of biofilm after attaining maturity. Cells can separate from the surface and migrate to colonise other substrates when the biofilm has reached maturity. Although the process of cell dispersal is complicated, processes of cell dispersal in biofilms have been identified thus far as seeding, erosion, and sloughing. The active process of cell dispersal, known as seeding or central hollowing, causes the biofilm to produce hollow holes as a result of the rapid discharge of huge numbers of cells or micro-colonies (Liu et al., 2024). Bacterial closeness and the sharing of mobile components within biofilms facilitate horizontal gene transfer and the spread of resistance determinants amongst strains and species (Madsen et al., 2012). These interactions contribute to increased antibiotic tolerance in biofilms. This high level of phenotypic tolerance observed in biofilm-associated cells, often described as 10-1,000 times lower susceptibility compared with planktonic counterparts, helps explain recurring and chronic infections (Sharma et al., 2019). The reduced drug penetration by the EPS, changed target expression, efflux activity, stress responses, and biofilm-specific regulatory programs are some of the overlapping processes that contribute to biofilm tolerance, according to molecular reviews and experimental research (Hall and Mah, 2017). As a result, biofilms contribute to a major health burden. According to clinical research, biofilms are common causes of persistent bacterial infections, including those related to prosthetic joints, cardiac devices, chronic wounds, and cystic fibrosis lung disease. Estimates indicate that at least 65–80% of persistent bacterial infections have a biofilm component (Bjarnsholt, 2013; Flores-Mireles et al., 2015).

These complications make it difficult for conventional antibiotics to manage biofilm-associated infections. The biofilm-associated phenotype necessitates the development of alternative therapeutic strategies that target key processes such as initial adhesion, quorum-sensing-mediated communication, extracellular matrix stability, and biofilm dispersion. This approach is essential since conventional antibiotics are predominantly designed to target planktonic bacterial cells and therefore exhibit limited efficacy against biofilm-embedded populations (Costerton et al., 1999; Høiby et al., 2010). Quorum-sensing and signalling inhibitors that interfere with coordinated virulence and biofilm development, together with enzymatic or chemical agents targeting the biofilm matrix, constitute promising anti-biofilm strategies. Furthermore, the incorporation of biofilm-dispersing molecules, phage-derived depolymerising enzymes, and nanotechnology-based delivery systems is engineered to improve antibiotic penetrations and retention within biofilms, offering a multifaceted approach to overcome biofilm-associated tolerance (Kaplan, 2010; Chan et al., 2013; Kalia, 2013; Saravanan et al., 2023). The current clinical guidelines underscore the necessity of integrating these strategies into routine medical practices. Biofilm pathology is becoming more widely acknowledged in clinical recommendations and consensus agreements, which also highlight the necessity of interdisciplinary approaches that integrate device management, antimicrobial stewardship, diagnostics, and innovative anti-biofilm treatments (Høiby et al., 2015). Computational and systems biology approaches are increasingly being applied to identify potential therapeutic targets and regulatory pathways involved in the formation and persistence of the biofilm (Miryala et al., 2019). Therefore, addressing biofilm-associated tolerance and its contribution to AMR necessitates coordinated innovation across clinical practice, materials science, pharmacology, and microbiology. The most practical way to lower persistent infections and slow the spread of resistance is to combine anti-biofilm modalities with improved stewardship and diagnostic tools. This review presents a comprehensive evaluation of the molecular mechanisms driving the biofilm-associated tolerance and explores 129 emerging therapeutic interventions including quorum quenching approaches, peptides, nanotechnology and computational drug discovery approaches. Additionally, it highlights the key challenges associated with clinical translation and outlines future directions for further facilitating the development of safe, effective and clinically feasible anti-biofilm therapies.

## 2 Mechanisms underlying biofilm-associated tolerance

Reduced susceptibility of bacteria to antibiotics in their sessile lifestyle and significantly contributes to the development of persistent, chronic illnesses. It is the result of a complex defence system that includes physical obstacles, physiological adaptations, coordinated communal efforts, and rapid genetic adaptation. The failure of conventional antibiotic monotherapy to eradicate mature biofilms is directly caused by the synergistic interaction of these diverse but connected mechanisms.

### 2.1 EPS matrix as a chemical and physical barrier

**Figure 1:**
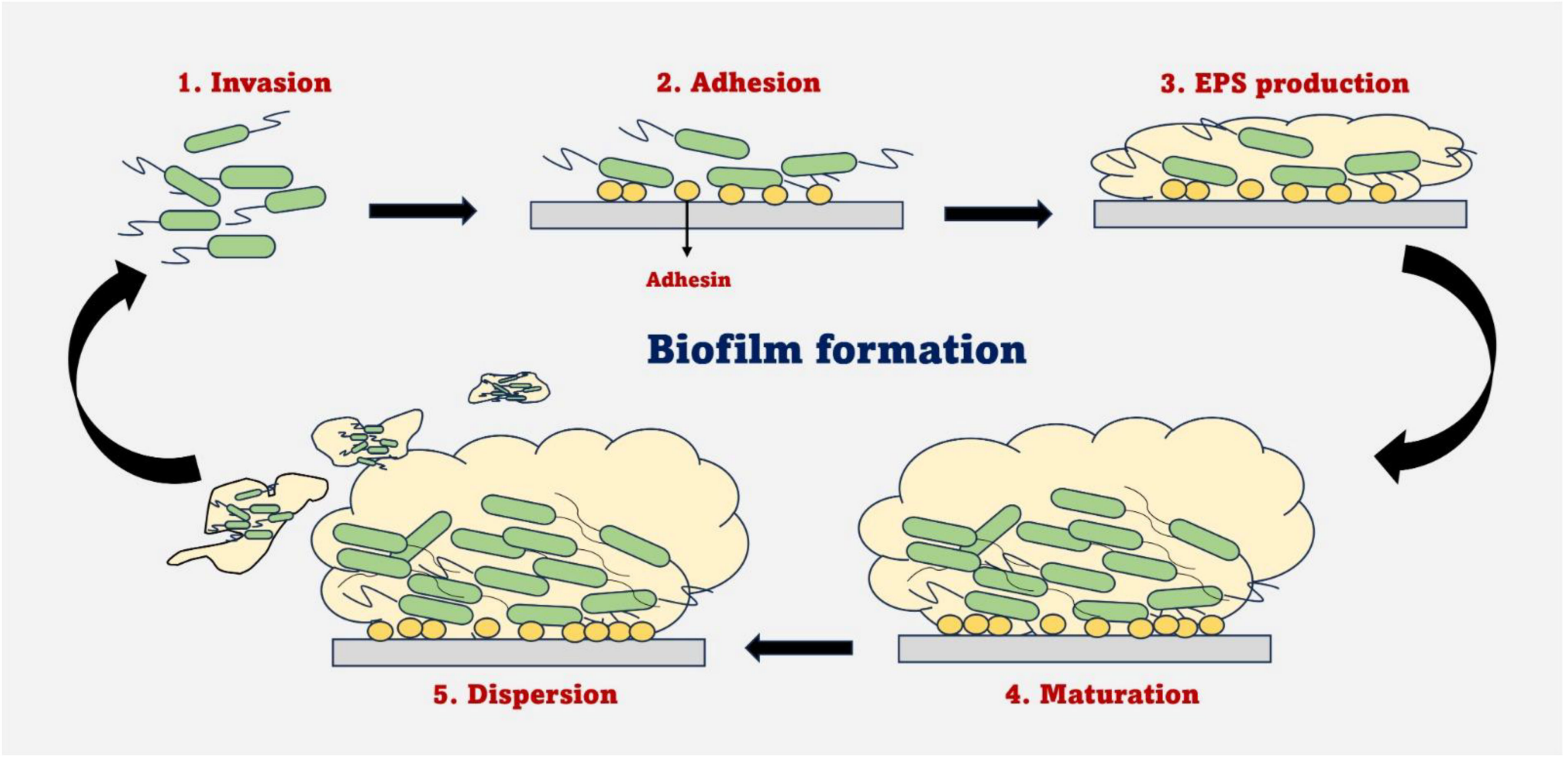
Schematic representation of the sequential stages of biofilm formation, including initial attachment of planktonic cells, irreversible adhesion, EPS production, maturation and eventual dispersion of cells to initiate new colonisation. This highlights key physiological and molecular transitions occurring during each stage.

EPS encapsulates organised multicellular populations known as biofilms, a schematic representation of the stages involved in biofilm formation is shown in **Figure 1**. The bacteria within biofilms exhibit a high level of phenotypic tolerance to antibiotics and immune responses. The EPS offers both structural stability and a protective barrier (Gunn et al., 2016). EPS plays a very important role in immobilizing biofilm cells and maintaining their proximity over an extended period, facilitating intense interactions such as cell-to-cell communication, horizontal gene transfer, and the development of synergistic microbial consortia. The matrix’s ability to retain extracellular enzymes allows the formation of a flexible external digestive system that can sequester, collect, and utilize dissolved and particulate nutrients that are transported through the aqueous phase. By preserving the components of lysed cells, including the DNA, the matrix often functions as the ultimate recycling reservoir and may even serve as a genetic repository. Biofilm biodiversity is further enhanced by chemical and nutrient gradients, which produce a vast array of heterogeneous microenvironments that strongly influence the bacterial physiology and susceptibility towards antibiotics, contributing to the phenotypic tolerance within biofilms (Flemming and Wingender, 2010).

### 2.2 Phenotypic tolerance and the adapted microenvironment

Even if the antibiotic successfully penetrates the outer EPS layer, they encounter a highly complex deeper layer within the biofilm. The three-dimensional structure of biofilm and high metabolic rates produce significant physicochemical gradients that produce a stratified and diverse microenvironment. The biofilm-bulk fluid interface with significant bacterial growth has an abundance of oxygen and nutrients. These substrates are rapidly depleted by metabolic activity, resulting in sharp gradients and isolated micro-niches with anoxia, nutrient limitation, and the buildup of acidic metabolic waste products (Hall and Mah, 2017). The main cause of phenotypic antibiotic tolerance is environmental stratification. The biofilm anoxic and oligotrophic core bacteria are compelled to use alternate metabolic processes and grow at a significantly slower rate. They become naturally tolerant to a variety of antibiotics because of this transition into a slow-growing or metabolically dormant phenotype. Additionally, the bacterial population rely on the communication systems to coordinate the adaptive responses to these stresses within the biofilm.

### 2.3 Quorum sensing

Quorum sensing is a complex cell-to-cell interaction system that allows bacteria to sense local cell density and coordinate gene expression (Miller and Bassler, 2001; Omwenga and Awuor, 2024). Within the biofilms, QS functions as an executive regulatory network that orchestrates the transition from a planktonic to a sessile lifestyle and thereby activates genes associated with virulence, stress tolerance, and in some cases AMR (Venkatesan et al., 2026). The process depends on the synthesis, accumulation, and detection of low-molecular-weight signalling molecules called autoinducers throughout the population, namely autoinducing peptides (AIP) in Gram-positive bacteria and acyl-homoserine lactones in Gram-negative bacteria.

When autoinducers accumulate beyond a critical threshold concentration, they bind to specific transcriptional regulators, triggering large-scale alterations in gene expression. QS-controlled genes include those involved in virulence factor secretion, EPS production, multidrug efflux pump activation, and biofilm maturation, all of which contribute to bacterial persistence and drug resistance (Flemming and Wingender, 2010). Through the QS-mediated coordination, an initially unstructured bacterial population is transformed into a highly organised and resilient biofilm community capable of mounting collective and anticipatory defence responses.

An important host-mediated regulatory mechanism influencing QS is the paraoxonase (PON) enzyme family, particularly paraoxonase-1 (PON1). Paraoxonases possess intrinsic lactonase activity, which enables them to hydrolyse the lactone ring of AHL molecules and disrupt QS signalling by preventing the accumulation of active autoinducers (Dong et al., 2001). As a result, paraoxonase enzymes function as quorum-quenching agents, attenuating bacterial communication without exerting direct bactericidal effects. Importantly, the reduced expression or activity of paraoxonase enzymes leads to enhanced QS signalling as diminished lactonase activity allows AHL molecules to persist and accumulate within the bacterial microenvironment (Teiber et al., 2003). Elevated QS activity under such conditions promotes proper biofilm formation and virulence expression, which helps to highlight the protective role of paraoxonase in host defence mechanisms. Several small-molecule compounds have been reported to interact with and inhibit paraoxonase activity. Inhibition of paraoxonase reduces AHL degradation and may indirectly amplify QS signalling. While PON inhibition enhances QS, targeting this interaction provides insights into regulatory modulation rather than therapeutic inhibition. To understand these phenomena at the molecular level, structure-based molecular docking studies have been widely used to analyse the interactions between selected compounds and the paraoxonase enzyme.

Docking studies provide insights into ligand binding orientation, affinity, and stability within the catalytic pocket of the enzyme, which helps in the identification of key residues involved in enzymatic inhibition (Kitchen et al., 2004). It revealed that effective paraoxonase inhibitors preferentially bind within the catalytic lactonase active site, forming stabilising interactions with residues essential for substrate recognition and hydrolysis. These interactions are mediated through hydrogen bonding with polar residues, hydrophobic interactions with nonpolar amino acids lining the binding pocket, and, in some cases, coordination with the catalytic metal ion, which is crucial for paraoxonase activity. Such binding modes restrict access of AHL substrates to the active site, thereby impairing lactonase-mediated QS signal degradation. Compounds exhibiting favourable docking scores demonstrated stable occupancy of the lactone-binding region, suggesting a strong inhibitory potential against paraoxonase. This inhibition is expected to decrease enzymatic AHL hydrolysis, facilitating autoinducer accumulation and subsequent activation of QS-regulated pathways. The docking-based interaction analysis establishes a mechanistic link between paraoxonase inhibition at the molecular level and functional modulation of QS signalling, providing a robust framework for understanding how enzyme–ligand interactions influence biofilm formation and bacterial virulence. Beyond this regulatory coordination, they develop specialised subpopulations that can further enhance the tolerance towards the antibiotics.

### 2.4 Persister cells

Biofilm communities also contain specialized phenotypic variants called the persister cells. They are tiny, specialized fractions that exhibit transient, non-inheritable, and multi-substrate multidrug tolerance and are one of the main characteristics of biofilm populations (Fisher et al., 2017). They are phenotypic variations that went into metabolic dormancy and lost their sensitivity to the deadly effects of almost all known antibiotics, rather than genetically resistant mutants. Environmental stress in the biofilm, such as oxidative stress or nutrient deprivation, may cause persister cell development, or it may be an emergent stochastic process (Niu et al., 2024). Complex molecular mechanisms, typically involving the induction of toxin-antitoxin (TA) systems, govern persister development. These systems’ “toxin” component usually works to inhibit vital cellular processes like translation or replication, which stops cell division and induces dormancy. The majority of metabolically active cells can be effectively eliminated by aggressive antibiotic therapy, but the persister population is unaffected. These cells can “resuscitate” and resume metabolic activity when the medication is stopped, which can result in biofilm repopulation and a clinical relapse of infection (Fisher et al., 2017). While these persister cells enable the survival of the bacterial population through metabolic dormancy, they also employ an active molecular mechanism to eliminate toxic components from the cell.

### 2.5 Efflux pumps

In addition to passive and physiological defence mechanisms, bacteria also employ energy-rich active transport systems to reduce intracellular accumulation of antibiotics. The most significant of these are membrane-embedded efflux pumps. These extrude a variety of toxic substances from the bacterial cytoplasm as molecular machines (Hall and Mah, 2017). These pumps prevent antibiotics from accumulating at their intracellular target sites and belong to several superfamilies, such as the Resistance-nodulation-division (RND) and ATP-binding cassette (ABC) family, Major facilitator superfamily (MFS), Small multidrug resistance (SMR), Multidrug and toxic compound extrusion (MATE) family and Proteobacterial antimicrobial compound efflux (PACE) family (Gaurav et al., 2023). The upregulation of efflux pump expression specific to biofilms is a crucial component of this mechanism. The genes encoding particular efflux pumps are substantially more expressed in biofilm-grown cells than in their planktonic counterparts, as several experiments have shown. Among these, RND-type efflux pumps are particularly significant against Gram-negative bacteria and have been extensively shown to contribute to reduced susceptibility within biofilms due to their broad substrate range and high transport efficiency. It has been demonstrated that *Pseudomonas aeruginosa* Mex-type efflux pumps are overexpressed in biofilms and play a major role in the high level of phenotypic tolerance observed in this form (Zhang and Mah, 2008; Kulandaivelu et al., 2026). Although RND efflux systems are the most studied in Gram-negative biofilm-associated tolerance, other efflux pump families also contribute significantly to antibiotic tolerance. For instance, MFS and ABC transporters have been associated with biofilm formation, virulence, and stress adaptation, whilst MATE and SMR systems contribute to the extrusion of specific antibiotics and antiseptics. The recently identified PACE is primarily linked to resistance against biocides and disinfectants, highlighting its emerging role in clinical settings (Gaurav et al., 2023; Du et al., 2018).This suggests that active efflux is an important adaptive mechanism specific to the social biofilm mode of lifecycle and offers effective and all-around defence against a wide range of antibiotics. Moreover, they also facilitate the rapid acquisition and dissemination of resistance through genetic exchange.

### 2.6 Horizontal gene transfer

The physical structure of the biofilm itself is a highly dynamic environment for HGT, acting as a hotspot for the quick evolution and spread of genes that confer antibiotic resistance (Hall and Mah, 2017). The biofilm habitat amplifies a number of HGT mechanisms. Conjugation is the transfer of plasmids and other mobile genetic factors by cell-to-cell direct contact and is greatly enhanced by physical proximity and close cell density in the matrix. Transformation is a sizable and stable reservoir of genetic data from dying cells, which makes up the eDNA component of the EPS matrix. Transformation is the process that naturally competent cells of the biofilm use to absorb the extracellular DNA. The safe and compact biofilm environment also facilitates the transfer of encapsulated genetic material within outer membrane vesicles (OMVs) and transduction (Grooters et al., 2024). The rapid spread of AMR genes throughout the population and significantly between species in polymicrobial biofilms is made possible by this elevated degree of gene transfer. The biofilm environment facilitates the development and spread of multidrug resistance, which poses a continuous and growing risk to public health. Consequently, the biofilm-mediated HGT not only accelerates the dissemination of multidrug resistance but also complicates the treatment outcomes, underscoring the urgent need for alternative anti-biofilm strategies.

## 3 Anti-biofilm therapeutics

**Figure 2:**
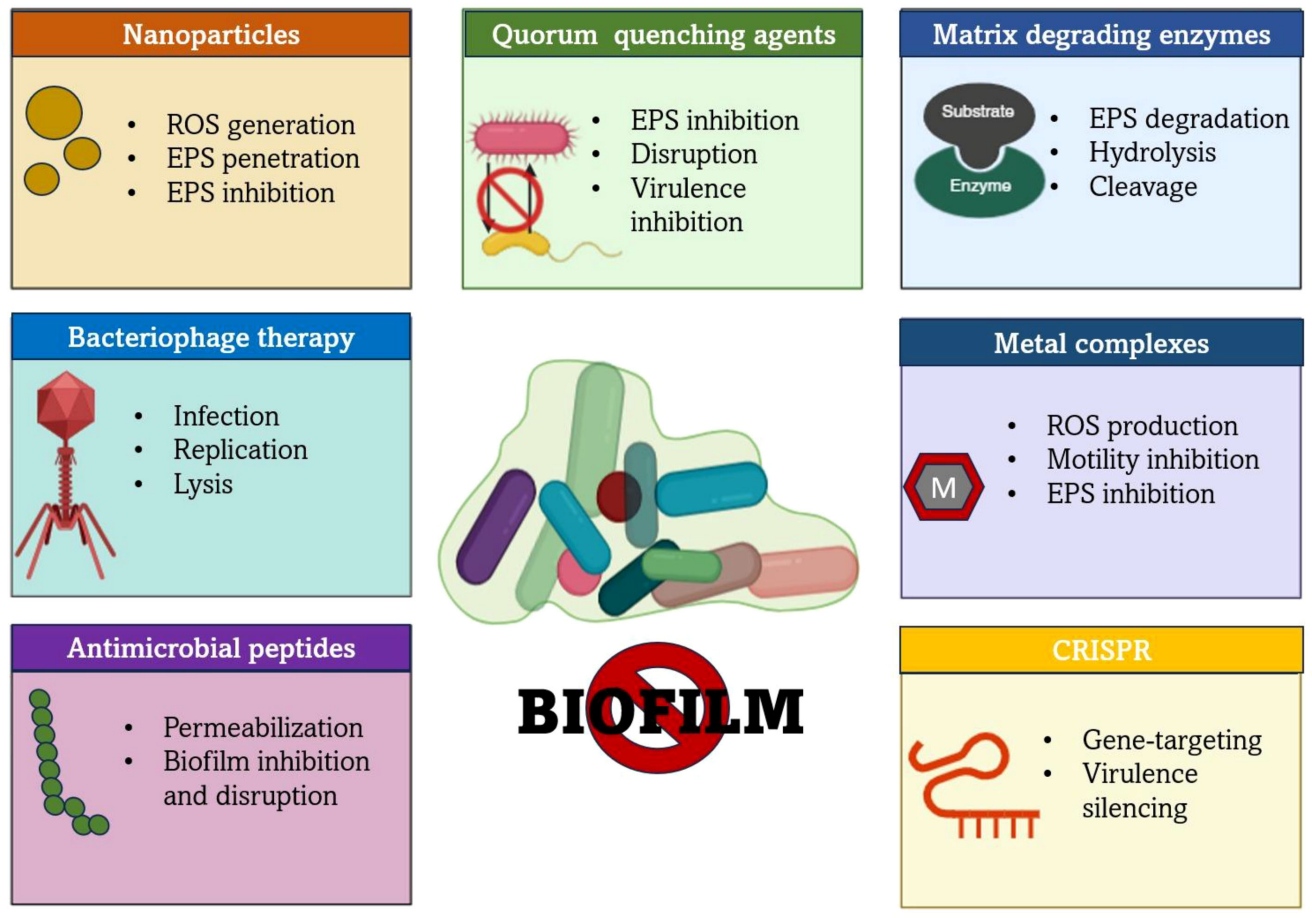
Overview of anti-Biofilm therapeutic strategies and their major mechanisms of action. This highlights how different approaches target various components of biofilm architecture, such as quorum sensing, EPS matrix, bacterial membrane and genetic regulation to inhibit the formation of biofilm.

The capacity of biofilms to withstand and endure extreme environmental conditions and subdue the host immune system has led to a need to investigate novel antibiofilm agents. Antimicrobial peptides (AMPs), efflux pump inhibitors (EPIs), quorum quenching agents, small molecule inhibitors, and nanomaterials are emerging biofilm control strategies that are gaining recognition for their ability to operate through several pathways and counteract resistance selectively (Hindieh et al., 2025). The antibiofilm therapies and their major mechanisms of action are illustrated in **Figure 2**.

### 3.1 Quorum quenching agents

As quorum sensing plays a major role in regulating biofilms, disruption of this *via* quorum quenching (QQ) has emerged as a promising antibiofilm strategy (Omwenga et al., 2023). QQ is a promising target for drug development since it increases the susceptibility of microbial biofilms to antibiotics and decreases the pathogenicity and virulence of bacteria (Subramani et al., 2025). N-acyl homoserine lactones lactonase is a QQ enzyme that hydrolyses the lactone ring of AHLs, which are important signalling bacterial quorum-sensing systems, reducing the effectiveness of the molecule (Dong et al., 2000). Both SsoPox W263I and GcL, two QQ lactonases that are selective for 3-oxo-C12-HSL and have a broad spectrum towards 3-oxo-C12-HSL and C4-HSL from the PLL and MBL families, suppressed the virulence-related characteristics of *P. aeruginosa* strain PA14 (Rémy et al., 2020; Vadakkan et al., 2020). *Alicyclobacillus acidoterrestris*, a thermoacidophilic bacterium, is the source of AaL, an MBL that exhibits a wide variety of substrates and a higher capacity to inhibit *Acinetobacter baumannii* biofilm development (Bergonzi et al., 2018). The AHL-lactonase produced by *Bacillus cereus* directly controls the production of virulence factors in *P. aeruginosa* (PAO1), including exopolysaccharide, biofilm formation, and pyocyanin (Paluch et al., 2020). The metallo-β-lactamase QQ enzymes LrsL effectively prevent *P. aeruginosa* from producing biofilms. Studies have shown that EPS inhibition by LrsL, a major biofilm component, aids in the formation of biofilm (Rehman et al., 2022). AHL acylase from *P. aeruginosa* breaks 3-oxo-C12-HSL and C4-HSL. Bacteria acquire N-octanoyl-L-homoserine lactone, an AHL associated with inter-species acylases in *E. coli*, by horizontal gene transfer of *AiiD* from *Ralstonia* (Lin et al., 2003). Reports suggest that oxidoreductases reduce the production of virulence factors by interfering with *S. aureus*’s capacity to synthesise autoinducing peptides (AIPs). This is a feasible method for interfering with the *S. aureus* QS system (Qazi et al., 2004). While QQ targets bacterial communications systems, nanotechnology-based therapeutics emerged as a physical and chemical strategy for directly disrupting the biofilm structure and viability. The mechanistic classification of anti-biofilm strategies targeting different components of biofilm architecture is summarised in **Table 1**, which has been systematically compiled busing a target level framework, including biological targets and mechanisms of action to provide a comprehensive and structured overview.

**Table 1:** Mechanistic classification of anti-biofilm strategies targeting different components of biofilm architecture.

Biofilm target level	Biological target	Mechanism of action	Therapeutic strategy	Examples	Pathogens	References
Cell-cell communication	Quorum-sensing signals	Signal degradation, interference, inhibition of virulence expression	Quorum quenching enzymes	LrsL; SsoPox W263I; GcL; AaL	*P. aeruginosa*, *A. baumannii*	(Rémy et al., 2020; Rehman et al., 2022)
Biofilm matrix	Polysaccharides, proteins, eDNA	Enzymatic hydrolysis and degradation of EPS	Matrix-degrading enzymes	Dispersin B; Proteinase K; Alginate lyase	*S. aureus, P. aeruginosa, E. coli*	(Barzkar et al., 2022; Al-Madboly et al., 2024)
Bacterial membrane and intracellular targets	Cell membrane, DNA, enzymes	ROS generation, membrane disruption, DNA damage, efflux inhibition	Metal complexes	Pd complexes, Ag(II) complexes, Cu(II) complexes	*S. aureus*, *E. coli*	(Baydeniz et al., 2024; Shobana et al., 2024)
Bacterial membrane and EPS interface	Membrane and EPS matrix	Electrostatic interaction, ROS generation, EPS penetration	Nanoparticles	AgNPs; CuO NPs; Zn NPs; Fe NPs	*E. coli, P. aeruginosa, S. aureus*	(Rani et al., 2024; Lopez Venditti et al., 2025)
Genetic regulation	Biofilm-associated genes	Gene targeting, cleavage, silencing	CRISPR-based systems	Gold NP-CRISPR; liposomal CRISPR-Cas9	*E. coli, P. aeruginosa*	(Saffari Natanzi et al., 2025)
Virulence and adhesion pathways	Motility, quorum-sensing pathways	Inhibition of adhesion, motility, biofilm formation	Phytochemicals	Curcumin; phloretin; fisetin	*S. aureus, P. aeruginosa, E. coli*	(Ghosh et al., 2025a; Mazumder et al., 2026)
Biofilm microenvironment	EPS permeability, drug diffusion	Heat-induced disruption, enhanced antibiotic release	Photothermal therapy	NIR-resonant polymer + daptomycin nanoplatform	*P. aeruginosa* *S. aureus*	(Zhou et al., 2025)

### 3.2 Nanotechnology-based approaches

Due to its superior mode of action, nanoparticle-based medicinal approaches are emerging as a substitute for conventional therapies. Metal oxide nanoparticles (NPs) and their composites have demonstrated remarkable antibacterial and antibiofilm properties (Saravanan et al., 2023). NP-biofilm interaction occurs through a sequential process involving transport of NPs to the biofilm interface, followed by the electrostatic attachment to the EPS matrix, and subsequent diffusion-driven penetration into the biofilm layer (Shkodenko et al., 2020). Additionally, electrostatic interactions between charged NPs and the predominantly negatively charged biofilm matrix play a key role in initial adhesion and retention. Further migration within biofilm is governed by physicochemical factors such as size, charge and hydrophobicity of the NPs. The production of copper oxide nanoparticles (CuO NPs) from the leaf extract of *Centratherum punctatum* showed strong antibacterial activity by preventing the biofilm formation in *P. aeruginosa*. Due to the eco-friendly synthesis and notable antibacterial and antibiofilm properties, CuO NPs are highlighted as excellent candidates for further exploration as antimicrobial agents (Rani et al., 2024). The antibiofilm activity of biocompatible silver nanoparticles (AgNPs) derived from leaf extracts of *Bridelia retusa*, *Glochidion lanceolarium* and *Semecarpus anacardium* was investigated. The produced nanoparticles were tested against the human pathogens *P. aeruginosa*, *E. coli* and *S. aureus* to determine their antibacterial and anti-biofilm properties. The findings showed AgNPs have a higher potential to function as anti-biofilm agents in biological applications, and the results of the study were promising (Mohanta et al., 2020).

Zinc nanoparticles (Zn NPs) biosynthesised from *P. aeruginosa* are effective against the tested pathogens in both planktonic and sessile forms. Zn NPs were found to inhibit up to 50% of yeast and *E. coli* biofilms and 80% of *S. aureus* biofilms (Lopez Venditti et al., 2025). The ability of green synthesised iron nanoparticles made from *Azadirachta indica* (neem) leaves to inhibit the growth of *S. aureus* was examined. The antibiofilm activity of the nanoparticle showed a dose-dependent pattern with MBIC_50_ and MBIC_90_ values of 1.56 mg/mL and 12.5 mg/mL (Mohammad Mohaidin et al., 2022). In a study, the aqueous leaf extract of *Orthosiphon stamineus* was used to synthesise palladium nanoparticles. According to the results of antimicrobial investigations, the newly synthesised nanoparticles showed improved antibacterial and antibiofilm activity against strains of extended-spectrum β-lactamase (ESβL) *S. aureus* and *E. coli* (Şahin et al., 2025). With an IC _50_ value of 15.25 ± 0.012 μg/mL, antibiofilm experiments demonstrated a concentration-dependent reduction of biofilm formation in the MSSA strain, whereas the control group demonstrated a weaker inhibitory effect (N et al., 2021). Despite their strong antimicrobial potential, they often benefit from the combination with agents that specifically degrade the biofilm matrices. The antibiofilm potential of various NPs is summarised in **Table 2**.

**Table 2:** Anti-biofilm activity and mechanisms of action of various nanoparticles against clinically relevant pathogens.

Nanoparticles	Biofilm inhibition (%)	Mechanism of action	Pathogens	Reference
Silver	Higher inhibition up to 96.9%	Attachment to the bacterial cell wall, thereby preventing adhesion to surfaces	*S. aureus* and *P. aeruginosa*	(Verduzco-Chavira et al., 2023)
Silver	30 - 97%	Suppression of biofilm genes and disruption of initial attachment	*A. baumannii*, *K. pneumoniae* and *P. aeruginosa*	(Elshaer and Shaaban, 2023)
Copper	59.4 - 88.94%	EPS inhibition, reduced cell wall hydrophobicity	*S. aureus* and *P. aeruginosa*	(Rahaman et al., 2025)
Copper	32 - 97%	Disruption of initial attachment and disruption of the biofilm matrix	*A. baumannii*, *K. pneumoniae* and *P. aeruginosa*	(Elshaer and Shaaban, 2023)
Zinc oxide	75.3 - 86.4%	ROS generation and disruption of biofilm architecture	*E. coli*	(Elabbasy et al., 2025)
Zinc	50 - 80%	Reactive nitrogen intermediates	*S. aureus* and *E. coli*	(Lopez Venditti et al., 2025)
Zinc oxide	58.18 - 67.3%	Size-dependent biofilm inhibition and biofilm penetration	*S. aureus and P. vulgaris*	(Mahamuni et al., 2019)
Palladium	Up to 72.73%	Reduction of microcolony and ROS	*S. aureus*	(N et al., 2021)
Titanium dioxide	70.67 - 80%	Biofilm inhibition without cell viability interference and possible ROS production	*S. aureus, P. aeruginosa*, and *E. coli*	(Altaf et al., 2021)
Titanium dioxide	71.85 - 79.96%	ROS generation and EPS inhibition	*S. aureus* and *E. coli*	(Bano, 2025)

### 3.3 Matrix degrading enzymes

EPS-degrading enzymes have been investigated in preventing the formation of biofilms and in eliminating those that have already been formed (Kim et al., 2023). Treatment with protein-degrading enzyme Proteinase K significantly reduced the formation of biofilms in clinical isolates of *S. aureus* by 90% in 13 out of 17 isolates. It further interrupted preformed biofilm in 14 out of 17 strains up to 80% (Sugimoto et al., 2018). Subtilisin, a serine protease, exhibits encouraging anti-biofilm activity. In a study, subtilisin treatment decreased the biofilm CFU of *Pseudomonas fluorescens* (Araújo et al., 2017). *Aggregatibacter actinomycetemcomitans* produces dispersin B, a broad-spectrum glycoside hydrolase that specifically targets poly-N-acetylglucosamine (PNAG), in the biofilm matrix that aids the majority of Gram-Positive and Gram-Negative bacteria to withstand environmental stress and antimicrobial drugs. This enzyme has been shown to disintegrate bacterial aggregations, disrupt existing biofilms and prevent the formation of biofilms and pellicles (Al-Madboly et al., 2024).

According to research, *P. aeruginosa* biofilm can be disrupted by purified alginate lyase from the marine *Pseudoalteromonas* bacterium by breaking down alginate in the EPS matrix. This further enhances the bactericidal activity of tobramycin, thereby exhibiting a synergistic therapeutic approach (Barzkar et al., 2022). Alpha amylase was found to be a potent antibiofilm agent against *Vibrio cholera* and *P. aeruginosa*. Amylase exhibited greater antibiofilm action against *P. aeruginosa* and *S. aureus* (Vaikundamoorthy et al., 2018). Cellulase and ceftazidime together have demonstrated strong antibiofilm properties by preventing the production of biofilms and disrupting existing biofilms of *P. aeruginosa* (Fischer et al., 2014). In addition to this, host-derived antimicrobial peptides have demonstrated efficacy in targeting biofilms.

### 3.4 Antimicrobial peptides

Antimicrobial peptides (AMPs) represent an important class of broad-spectrum antimicrobials, ranging in molecular weight from 1 to 5 KDa and containing 5 to more than 100 amino acids, most of which are L-form. Because of their function in the immunological response, AMPs are commonly known as cationic host defence peptides (George et al., 2025a, b). They are frequently cationic in nature. AMPs have replaced traditional antibiotics because of their diverse target sites and non-specific mode of action, which decreases the likelihood of resistance development (Naha and Ramaiah, 2023). The strong anti-biofilm effect of AMPs can prevent biofilm formation in its early phases of development in both clinically isolated and multidrug-resistant bacterial biofilms (Yasir et al., 2018). Several AMPs have been reported to interfere with microbial adhesion and quorum-sensing pathways, thereby disrupting the establishment of biofilm. Significant antimicrobial efficacy against *P. aeruginosa* has been demonstrated by Denovo’s 1037 and LL-37, a human AMP. It influenced the QS system, decreased swimming and swarming motilities, increased twitching motility, and downregulated the genes necessary for biofilm formation (Overhage et al., 2008; de la Fuente-Núñez et al., 2012). The human-derived human β-defensin 3 (hBD-3) demonstrated antimicrobial action by targeting the genes *icaA, icaD*, and *icaR* in *Staphylococcus epidermidis* (Zhu et al., 2013).

The *Calliphora vicina* maggots, which live in habitats highly polluted by biofilm-forming microorganisms, create an AMP complex that has anti-biofilm action. Strong cell-killing and matrix-destroying properties are demonstrated by the complex against human pathogenic antibiotic-resistant *E. coli*, *S. aureus* and *A. baumannii* biofilms. Additionally, the complex is reported as non-toxic to human immune cells, providing further advantage in employing this in antimicrobial treatments (Gordya et al., 2017). Research evaluated potential β-lactamase inhibitors from antimicrobial peptide mutants with improved antibacterial potency (~7-16%) compared to their parent peptides. Using extensive knowledge-based and machine-learning techniques, the study tested five peptides and their mutations according to physicochemical and pharmaco-immunogenic characteristics. HFIAP-1_M5 (L33K-W7C-N34C) was identified by molecular docking analyses as a possible inhibitor candidate. Based on intermolecular interaction profiling, it was expected to inhibit around 82% of all the examined extended-spectrum β-lactamases in *E. coli* targets (George et al., 2025a). Recently, bacteriophage-based therapeutics have gained attention in addition to peptide-based approaches.

### 3.5 Phage therapy

Phage therapy has emerged as a promising strategy in combating biofilm-associated infections. Using different bacteriophage components, phage-based therapy may fight biofilm in many ways. Phages infect bacteria and are purely host-specific viruses that rely on their host to replicate. Since the discovery of antibiotics, research on phages and phage treatment has steadily returned, despite the decline in new antibiotic discoveries and the rise in AMR in recent years (Chang et al., 2022). Strong antibacterial action was demonstrated by bacteriophages from the *Siphoviridae* and *Myoviridae* families that were isolated from sewage samples. They exhibit encouraging anti-biofilm action against the tested isolates of *Enterococcus faecalis* (*E. faecalis*) that generate robust biofilms. In comparison to the control, the enterococcal phages decreased the preformed biofilms to a range of 71.0–80.0% and 38.02–45.7%, respectively. These phages are attractive candidates for use in preventing and treating *E. faecalis* infections linked to biofilms because of their promising antibiofilm activity (El-Atrees et al., 2022).

The biofilm mass was successfully eliminated by applying the phage to biofilms formed on the coverslip and Foley silicon catheter biofilm models. In order to investigate how intact biofilm architecture affects phage predation, the biofilms were broken apart. When the phage was incubated for 24 hours, the number of viable cells was reduced by 0.6 to 1.0 orders of magnitude. Bacterial biofilms may be disrupted and inhibited by the endolysin that the phage genome encodes. Furthermore, there were no genes in the genome that encoded lysogeny, virulence factors, toxins, or antibiotic resistance. For the treatment of urinary tract infections brought on by biofilm-forming MDR and XDR UPEC strains, lytic phage 590B may therefore be a useful alternative to antibiotics and might be utilised in phage cocktails (Chaudhary et al., 2022). Enterotoxigenic *E. coli* (ETEC)-phage-TG and BC-VP, two bacteriophages isolated from freshwater lakes, were assessed for their ability to combat enteropathogenic and enterohemorrhagic *E. coli* biofilms. The biofilm production on the polystyrene surface was inhibited and destroyed by the phage (ETEC)-phage-TG at 25.43% and 11.4%, respectively. By 37.87% and 39.21%, (ETEC)-phage-TG disrupted biofilm formation on the stainless-steel surface after 24 and 48 hours, respectively. In addition to inhibiting and destroying biofilm on polystyrene surfaces by 54.57% and 26.36% at 24 and 48 hours, BC-VP also inhibited biofilm on stainless steel by 33.21% and 43.94% at 24 and 48 hours. Together, these results highlighted the higher efficacy of these phages on antibiofilm therapies (Archell et al., 2025). Besides biological approaches chemical engineered metal complexes have also shown promise in antibiofilm properties.

### 3.6 Metal complexes

Employing metal complexes to combat biofilm-related illnesses has grown in recent years. Several mechanisms of action are demonstrated by these complexes at the molecular level, including cell permeability of microbial envelope, release of reactive oxygen species (ROS) or nitrogen (RNS), disruption of DNA, membrane proteins, EPS and inhibition of enzymes (Olar et al., 2022). Complexes based on palladium were investigated as NorA inhibitors. Without producing cytotoxicity, QSL_Pd5A demonstrated to be a potent efflux pump inhibitor that resensitised *S. aureus* to ciprofloxacin. Biofilm development was significantly decreased in a dose-dependent manner by QSL_Pd5A sub-MIC treatments, with higher doses producing an inhibition of up to 99% (Shobana et al., 2024). At sub-MIC concentrations, the synthesised zinc(II)-sorbate complex demonstrated significant antibiofilm potential by lowering biofilm adhesion to 36.4% in *B. cereus* and 42.7% in *E. coli* isolates (Abousaty et al., 2024). In another study, the π-conjugated Azoate-based Cu(II) complex with ligand 4,4’-((1*E*,1’*E*)-(naphthalene-1,5-diylbis(azaneylylidene))bis(methaneylylidene))bis(benzene-1,2-diol) showed the highest efficacy, suppressing Methicillin-Resistant *Staphylococcus aureus* (MRSA) biofilms by 95% at 64 µg/mL. Additionally, there was a 50% decrease in Methicillin-Susceptible *Staphylococcus aureus* (MSSA) biofilms. These findings demonstrate the good efficacy of copper complexes against biofilm-associated resistant infections caused by *S. aureus* (Baydeniz et al., 2024). Beyond these several other approaches, including phytochemicals, CRISPR-based therapeutics and photothermal therapies are being explored in effectively controlling biofilm.

### 3.7 Other approaches

Numerous studies suggest the potential and higher efficacy of antimicrobial and antibiofilm properties of phytochemicals. In a study, the three most potent flavonoids, curcumin, phloretin, and fisetin, inhibited the production of biofilms by *A. baumannii* in a dose-dependent manner (Ghosh et al., 2025a; Mazumder et al., 2026a). Additionally, the most potent flavonoid, curcumin, had stronger antibiofilm action than gallium nitrate, a well-known biofilm inhibitor. Curcumin suppressed *A. baumannii* surface motility and pellicle production. Curcumin, interestingly, has also shown antibiofilm efficacy against mixed cultures of *C. albicans* and *A. baumannii* (Raorane et al., 2019). In recent years, CRISPR-nanoparticle combination techniques have demonstrated encouraging results. By boosting target specificity, cellular absorption, and controlled release in biofilm settings, nanoparticles can improve the delivery of CRISPR. According to recent research, liposomal CRISPR-Cas9 formulations may reduce *P. aeruginosa* biofilm biomass by more than 90% *in vitro*, and gold nanoparticle carriers can boost editing efficiency by up to 3.5 times when compared to non-carrier systems. Additionally, these hybrid platforms allow for the co-administration of antibiotics, resulting in enhanced biofilm breakdown and synergistic antibacterial effects (Saffari Natanzi et al., 2025).

As a commonly used genome engineering tool, CRISPR Cas9 can play a crucial role in reshaping strategies to counteract antibiotic resistance. Additionally, it can play a major role in specifically disrupting the genes that cause biofilm development (Pandey and Vavilala, 2024). Additionally, photothermal treatment (PTT) surfaced as a successful biofilm removal method. But the hazards of injury to healthy tissues and biofilm recalcitrance are increased by the insufficient bacterial ablation in low-temperature PTT and the local high temperature of PTT, respectively. Heat-sensitive liposomes, conjugated polymers that respond to near-infrared light, and the antibiotic daptomycin for the removal of biofilms make up the synergistic nanoplatform. In order to destroy biofilm, the heat produced by conjugated polymers driven by 808 nm light changes the permeability of biofilms and causes the local release of antibiotics. For as long as five days, the nanoparticles may successfully stop the formation of biofilms and show biofilm dispersion activity. Because of this, this conjugated polymer-based nanoplatform provides a dependable way to remove biofilms from titanium alloy implants and shows promise for use in drug-resistant clinical settings (Zhou et al., 2025). In addition to experimental approaches, computational strategies further enhance antibiofilm drug discovery.

## 4 Computational approaches

**Figure 3:**
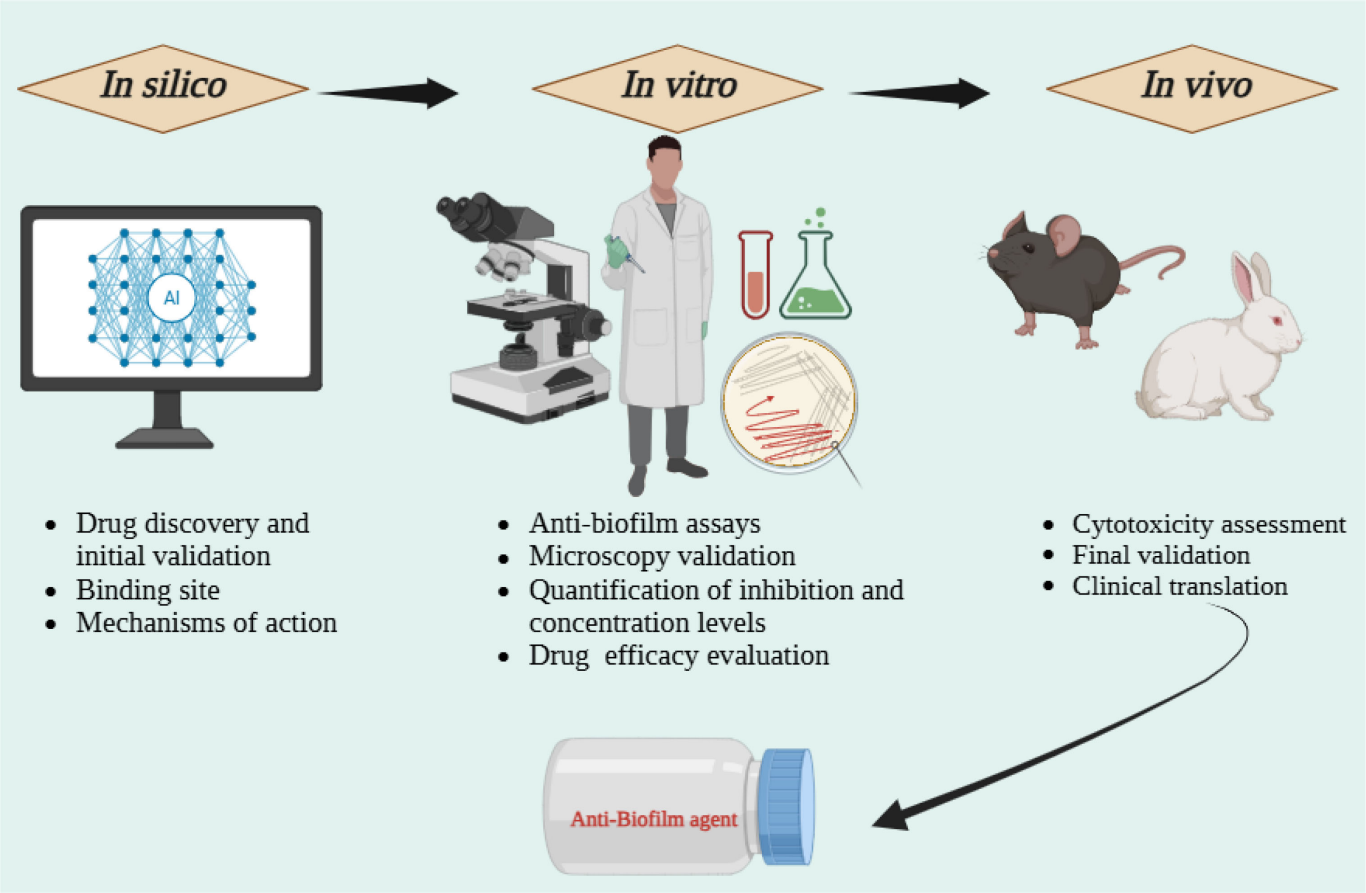
Overview of computational strategies employed in anti-biofilm research. This summarises the overall process of anti-biofilm drug discovery from computational modelling for optimisation, to *in vitro* experimentation for evaluation, and *in vivo* experiments for validation.

Computational (*in silico*) methods have developed from unique virtual screens into integrated pipelines that produce precise simulations, robust AI/ML design, and reliable systems-level models (Ghosh et al., 2025c). In the context of anti-biofilm strategies, these approaches extend beyond hit identification to include prioritisation, interpretation and guidance for the experimental validation and clinical translation as illustrated in **Figure 3**. The key computational approaches, along with their advantages and limitations, are summarised in **Table 3**.

**Table 3:** Overview of computational approaches in anti-biofilm therapies, highlighting their working principle, advantages and limitations.

Approaches	Principle	Advantages	Limitations	Reference
Molecular docking and dynamics simulations	Predicts protein-ligand binding to using scoring functions; dynamics simulates time-dependent stability and interactions of complexes under near-physiological conditions	Rapid screening of inhibitors Structural insights into binding Early ADMET filtering	Rigid docking lacks realism Limited experimental validation	(Li et al., 2025)
Artificial intelligence and machine learning	Utilizes statistical and deep learning models to classify, predict and design	Enables *de novo* design Predicts drug synergy Multi-objective optimization	Dataset bias Limited toxicity prediction	(Shah et al., 2024)
Bioinformatics-guided genome engineering	Applies genome analysis, host prediction algorithms, and comparative genomics to identify and engineer phages	Predicts host specificity Enables genome editing Improves biofilm penetration Safety screening possible	Regulatory hurdles Incomplete host prediction Limited clinical translation	(Yutin et al., 2021)
Computational enzyme engineering	Applies protein modelling and directed evolution to design enzymes with improved stability, activity or specificity	Rational enzyme optimisation Improves EPS degradation High-throughput screening Enhances catalytic efficiency	Stability *in vivo* is still limited Delivery challenges Scale-up issues	(Adelusi et al., 2022)
Systems biology and network modelling	Employs network analysis, metabolic reconstructions to simulate polymicrobial biofilms interactions	Captures complex interactions Identifies key regulatory hubs Predicts system-level response	Requires large datasets Limited clinical validation Complex modelling	(Seçilmiş et al., 2020)
Multi-omics data integration	Integrates genomics, transcriptomics, proteomics, and metabolomics to identify disease signatures and therapeutic targets	Enables personalised therapy Identifies molecular modulus Improved biological insight Better target prioritisation	Data heterogeneity Difficult clinical translation Limited prospective validation	(Li et al., 2022)
Simulation of biofilm microenvironment	Uses reaction diffusion models and hybrid simulations to model gradients and spatial heterogeneity in biofilms	Predicts diffusion barriers Guides drug delivery Captures spatial heterogeneity	High computational cost Needs experimental validation	(Wang et al., 2025)

### 4.1 Molecular docking and dynamics simulations for quorum-sensing inhibitors

Earlier studies highlighted structure-based screening as a rapid method for nominating quorum-sensing inhibitors (QSIs) and efflux pump inhibitors (EPIs). But many docking studies were criticised for relying on rigid receptors and omitting membrane context, absorption, distribution, metabolism, excretion, and toxicity (ADMET) filters (Rathi et al., 2020). Experimental follow-up for many *in silico* hits was not frequent, leaving it uncertain which predictions survive biochemical and cellular testing. Representative *in silico* QS screening work used docking and short MD to triage natural-product hits against *LasR/PqsE* and related regulators (Chaieb et al., 2022). More recent studies have moved towards integrated pipelines that pair docking with longer molecular dynamics simulations, binding-free energy calculations, and early ADMET filtering to reduce false positives and improve prioritisation for bench assays (Chaieb et al., 2022; Duffey et al., 2024). This evolution is visible as an early optimism about high-throughput docking, with critiques about the lack of realism and validation, and a more recent adoption of membrane-explicit MD and thermodynamic scoring to strengthen translational relevance. While these docking and dynamics simulations provide structural insights into the ligand interactions with the targets, the increasing complexity of drug discovery has driven the adoption of artificial intelligence and machine learning approaches to accelerate the process.

### 4.2 Artificial intelligence and machine learning for AMPs and synergy prediction

Recent studies demonstrated that ML has significantly accelerated AMP discovery from classification models that discriminate AMP vs non-AMP, to deep generative models that propose *de novo* sequences, but they repeatedly call out dataset bias, inconsistent activity assays, and insufficient toxicity/stability modelling (Wan et al., 2024). More recent papers and reviews show progress in generative models (transformers, VAEs) that include multi-objective constraints (potency + haemolysis/stability), and benchmarked datasets for standardised evaluation. Similarly, reviews of ML for drug synergy prediction summarise algorithms (similarity-based, network + deep learning) and emphasise the need for large, standardised combination screens to train robust predictors (Torkamannia et al., 2022). In short, ML methods are stronger at *in silico* filtering and ideation than at guaranteeing *in-vitro* success, but the field is actively addressing this *via* curated datasets and toxicity-aware objectives.

### 4.3 Bioinformatics-guided phage selection and genome engineering

Earlier studies have shown the rise of computational tools that predict phage hosts and flag undesirable genes (Andrianjakarivony et al., 2022). Early pipelines focused on lytic phage identification and safety screening, whilst many recent studies describe how ML and comparative genomics can guide targeted engineering, such as swapping or altering depolymerase/receptor binding proteins, to broaden the host range or improve biofilm penetration (Doud et al., 2025). Computational host prediction enhances candidate selection, but practical barriers (regulatory pathways, immune interactions *in vivo*) still slow translation. Early reviews flagged unpredictable host range and slow empirical selection, whilst subsequent computational host-prediction and RBP analysis pipelines have partially solved rapid matching of phages to isolates, and genome editing approaches have been used to add EPS-degrading functions, yet clinical adoption requires standardised safety/efficacy data. In addition to this, computational approaches are being increasingly applied to the rational design, development and optimisation of matrix-degrading enzymes.

### 4.4 Computational enzyme engineering for matrix-degrading enzymes

Advances in computational protein engineering have enabled the rational design of more stable and active enzymes (Shao et al., 2023). For EPS-degrading enzymes (DNases, glycosidases, proteases), the main translational issues are activity and stability in complex, protease-rich and variable pH environments. High-throughput computational directed-evolution frameworks (e.g., EnzyHTP) that automate variant generation and triage have produced variants with improved thermostability/catalysis in model systems, thereby addressing the stability gaps highlighted in earlier enzyme-centric reviews. Earlier studies emphasised instability and poor *in vivo* performance. EnzyHTP and similar computational plus directed evolution pipelines have demonstrably improved hit rates for stabilised enzyme variants, though delivery, immunogenicity, and scale-up still require engineering solutions.

### 4.5 Systems biology and network modelling of polymicrobial biofilms

Many studies that focused on systems approaches show that single-species studies miss emergent properties of polymicrobial biofilms (metabolic cross-feeding, quorum-sensing cross-talk, HGT hotspots). Agent-based models (ABMs) and metabolic/network reconstructions are essential tools for capturing spatiotemporal dynamics (Nagarajan et al., 2022). Recent studies combine time-series transcriptomics with ABMs to simulate community transitions and to identify conserved network nodes amenable to multi-target therapy. The systems biology network analysis, including the protein-protein interaction and gene network analysis, can be employed to identify key hub genes governing the adhesion and biofilm development (Naha et al., 2022). The literature shows steady progress in integrating data modalities, though validation in human infection samples remains limited. Foundational reviews have highlighted standardised longitudinal multi-omics datasets and better integration tools; some groups now produce time-series multi-omics + ABM studies that begin to close this gap, yet prospective clinical validation of model-derived interventions is still scarce. To capture the biological complexity, multi-omics approaches have emerged as a powerful tool in understanding host-pathogen interactions and personalised therapeutics.

### 4.6 Multi-omics data integration for personalised anti-biofilm therapy

Previous studies of multi-omics integration emphasise precision therapy but call out heterogeneity, batch effects, and the difficulty of linking omics signatures to actionable therapy recommendations (Muller et al., 2024). Recent advances, including intermediate-integration frameworks like MintTea, standardised evaluation protocols, increase robustness and interpretability, enabling the identification of multi-omics modules associated with disease. However, applying these pipelines to acute biofilm infections is challenging because accessible sampling of the infection microenvironment is often invasive and time-sensitive. While method papers provide integration frameworks, few clinical trials have prospectively used multi-omics modules to select tailored anti-biofilm regimens. This remains the primary bottleneck for personalised anti-biofilm therapy.

### 4.7 Simulation of biofilm microenvironment

Both classic and recent studies emphasise that the microenvironmental heterogeneity (oxygen and pH gradients, antibiotic diffusion limitation) explains many failures of *in vitro* active compounds in structured biofilms. Agent-based and continuum reaction–diffusion models capture these gradients and predict pockets of tolerance (Nagarajan et al., 2022; Pechaud et al., 2024). The next step has been hybrid models that couple spatial gradients with molecular responses at single-cell resolution; these hybrid simulations improve the prediction of where persisters will persist and how delivery systems need to be designed to reach them. Computation cost and parameterisation for patient-specific simulations remain practical barriers. Earlier models lacked mechanistic coupling to molecular response data; recent hybrid spatial-molecular models partially fill this gap and provide actionable predictions for dosing and delivery system design. Yet computational expense and a need for patient-level parameter estimates limit clinical application.

## 5 Challenges to anti-biofilm therapies

Although numerous antibiofilm strategies have been developed, several challenges limit their clinical application. This includes the challenges arising from the complex architecture of the biofilms, polymicrobial interactions, and persistence mechanisms.

### 5.1 Challenges against polymicrobial biofilms

Compared to the single-species biofilms, polymicrobial biofilms exhibit higher tolerance to antibiotics. The extracellular matrix (ECM) of polymicrobial biofilms, composed of polysaccharides, proteins, and extracellular DNA secreted by different organisms, makes it resistant to therapeutic approaches (Liu et al., 2024). Biofilms also facilitate horizontal gene transfer, conjugation, transformation, and phage-mediated transduction, enabling the exchange of antibiotic resistance genes within and between species in mixed communities, enabling the rapid evolution of resistance in polymicrobial infections (Nithyanand et al., 2025). Antibiotic tolerance is increased by interspecies interactions, which shape the biofilm architecture, metabolism, and virulence gene expression. For example, *C. albicans* protects bacterial partners within the polymicrobial biofilms, whilst bacteria may secrete molecules that enhance the tolerance of neighbouring species. Consequently, these interactions enhance the resilience and persistence, making single-agent therapies insufficient (Anju et al., 2022). Persister cells do not show resistance to the drug but show extreme tolerance since they are metabolically inactive, which makes the drug target inactive as well. When active cells are killed after a course of therapy, the persister cells can switch to the active state, which causes the infection to relapse. Therefore, the anti-biofilm therapies must target these dormant persister cells (Liu et al., 2024; Nithyanand et al., 2025).

### 5.2 Challenges posed by regrowth and resistance

The ECM and its associated factors support the regrowth by slowing antibiotic penetration and chemically interacting with antibiotics, reducing effective concentrations within the biofilm. The physical diffusion barriers created by the ECM contribute to biofilm recalcitrance. This prevents antibiotics from penetrating deep into the biofilm, and simultaneously causes matrix-mediated sequestration, where the ECM traps or chemically neutralises antibiotics, thereby lowering the effective concentration that reaches the target cells (Uruén et al., 2020).

Quorum sensing (QS) in biofilm modulates the resilience and post-treatment regrowth. QS influences matrix production, virulence factor expression, and resistance determinants. Disrupting QS can increase susceptibility but may not fully prevent regrowth without additional interventions (Hall and Mah, 2017). Regrowth and resistance persist even after non-antibiotic strategies such as colistin-based combinations and meropenem regimens. Adjunctive strategies such as anti-persister compounds, matrix-degrading enzymes, and quorum-quenching agents may help reduce regrowth by targeting multiple recalcitrant mechanisms in concert with antibiotics, their clinical translation remains limited (Soares et al., 2020).

### 5.3 Challenges in experimental models

**Figure 4:**
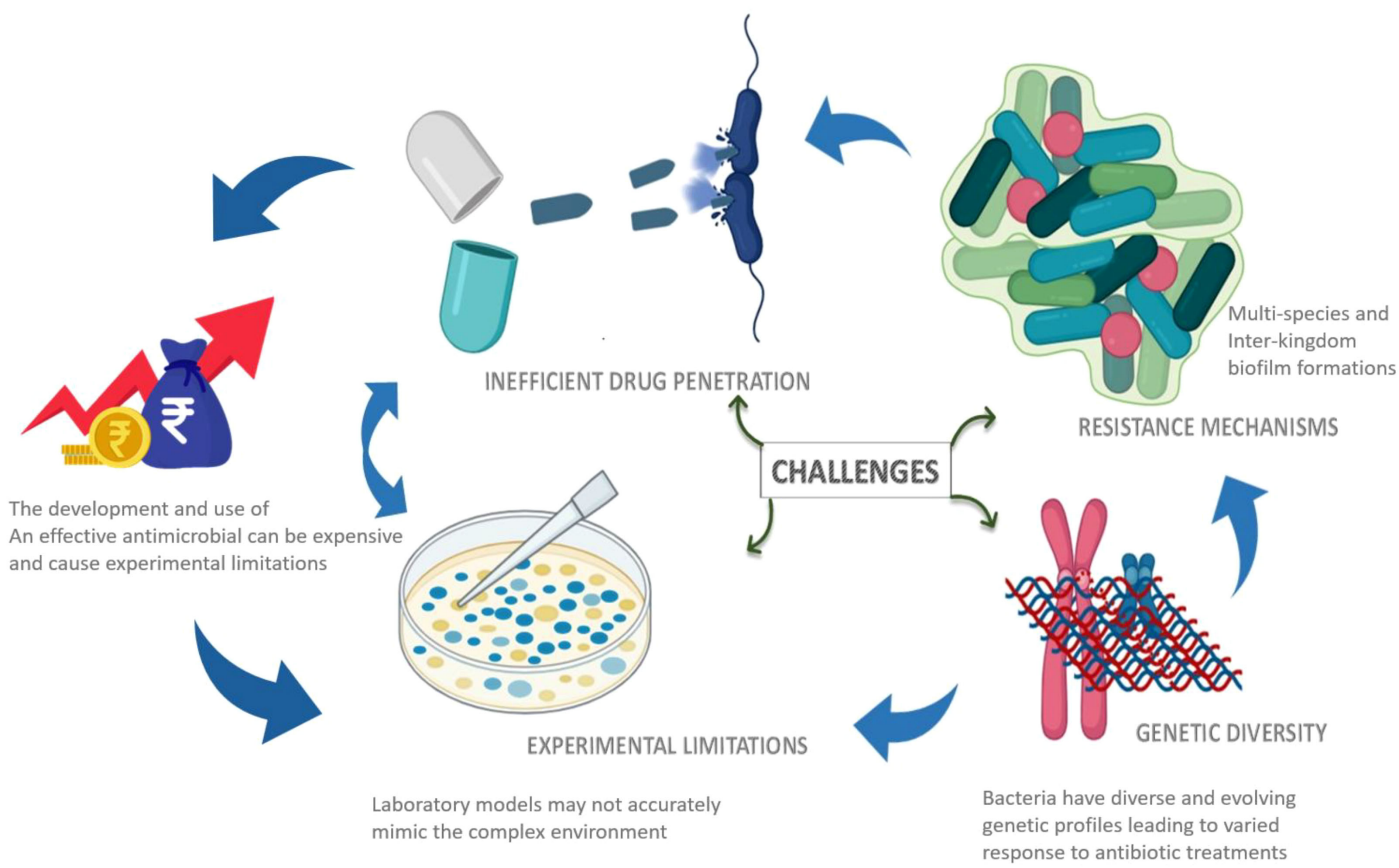
Major challenges associated with anti-biofilm therapy. This highlights the critical barriers, including limited penetration of antimicrobials, multi-species biofilm, genetic diversity and adaptive resistance mechanisms associated with biofilm formation.

Biofilms often comprise multiple species with diverse EPS composition and internal microenvironments, which leads to variable drug exposure and outcomes across models​. Therefore, antibiotics often show non-uniform diffusion through the matrix. Deeper layers experience nutrient limitation, reducing drug efficacy and enabling subinhibitory exposures that can foster persistence and adaptive responses (Lebeaux et al., 2014; Crivello et al., 2023; Lorenz et al., 2023). Mechanisms include impaired diffusion, matrix interactions, biofilm-specific gene regulation, efflux, and quorum-sensing–driven tolerance. The relative contribution of each mechanism depends on antibiotic class and biofilm context, thereby complicating universally applicable strategies. *In vitro* models fail to fully recapitulate clinical complexity. Multispecies and interkingdom biofilms improve realism but reduce throughput and standardisation, challenging interpretation and translation to therapy. Different quantification methods (biomass assays, CFU, viability imaging) can yield divergent impressions of efficacy, hinder cross-study comparisons and limit the ability to draw robust conclusions about antibiofilm interventions. **Figure 4** highlights the various challenges in antibiofilm therapies.

### 5.4 Clinical translational challenges in low- and middle-income countries

The targeted therapies have shown considerable promise in experimental as well as *in silico* studies, but the implementation in real-time clinical settings remains challenging, especially in the low- and middle-income countries (LMICs). One of the major barriers in clinical translation is the high cost and manufacturing complexity of advanced therapeutics such as AMPs, phage cocktails, and engineered enzymes, as they require strict quality control, optimised production and logistics, which is often difficult to maintain in health care with limited resources (Leptihn and Loh, 2022; Yarahmadi et al., 2025). The lack of regulatory frameworks for the antibiofilm strategies is another limitation. In LMICs, the regulatory agencies are still developing in order to assess efficacy, safety and quality of the complex therapeutics such as CRISPR-based antibiofilm therapies, bacteriophage therapy and nanomedicine. The clinical translation is also complicated by limited diagnostic tools, such as advanced molecular and genomics profiling to detect biofilm formers and targeted therapy.

## 6 Future perspectives

Future directions in combating biofilm-associated antimicrobial resistance should prioritise adaptive, precision and clinically translatable strategies. Recent advances in single-cell omics and spatial transcriptomics enable high-resolution mapping of biofilm heterogeneity, allowing identification of novel therapeutic targets (Dar et al., 2021). Integrating these datasets with AI-driven predictive modelling can facilitate rational design of combination therapies that simultaneously target quorum sensing, persister cells and metabolic gradients. The development of stimuli-responsive nanotherapeutics, capable of controlled drug release in response to pH, enzymes and redox conditions within biofilms, represents a promising avenue to overcome diffusion barriers. In parallel, synthetic biology approaches, including engineered probiotics and programmable microbial systems, are emerging as innovative tools to disrupt pathogenic biofilms and restore microbiome balance. Another critical area is the advancement of anti-virulence therapies, which impose lower selective pressure compared to conventional antibiotics, potentially reducing resistance evolution (Dehbanipour and Ghalavand, 2022). Moreover, translating these innovations requires the establishment of standardised regulatory frameworks and clinically relevant infection models, particularly for polymicrobial biofilms. Bridging the gap between bench and bedside will depend on interdisciplinary collaboration, scalable manufacturing, and global accessibility, ensuring that next-generation antibiofilm therapies are both effective and widely deployable.

## 7 Conclusion

The complex resistance mechanisms and exceptional flexibility of the biofilms make it one of the most difficult challenges in contemporary medicine. Physical barriers, phenotypic variability, quorum sensing, efflux mechanisms, and persister cells work in concert to give biofilms an impressive resistance to common antibiotics. Pathogens can become re-sensitised to antibiotics through current therapeutic developments in metal complexes, phage treatment, antimicrobial peptides, matrix-degrading enzymes, quorum quenching agents, and nanotechnology-based methods; they present an intriguing approach to compromising the integrity of biofilms. Computational and AI-driven frameworks have significantly increased our ability to predict molecular targets, optimise drug design, and model biofilm dynamics, suggesting a shift towards precision-driven anti-biofilm therapy. However, it is still challenging to effectively implement these findings in clinical areas due to the difficulty in reproducing *in vivo* biofilm diversity, polymicrobial interactions, and host-pathogen interactions. This integrated approach will be essential in developing hybrid therapies that incorporate enzymatic, nanomaterial and quorum-quenching methods in order to disrupt mature biofilms. The use of customised anti-biofilm treatments, backed by multi-omics data and systems biology modelling, is the way forwards, considering the future of precision infection control. To transform these novel approaches into workable, patient-centred solutions against biofilm-mediated antimicrobial resistance, it is important to advance translational research, standardise *in vitro* and *in vivo* biofilm models, and promote cooperation between scientific and clinical domains.
